# Prevalence and Predictors of Premature Graying of Hair Before the Age of 30: A Cross‐Sectional Study in Saudi Arabia

**DOI:** 10.1111/jocd.16627

**Published:** 2024-10-09

**Authors:** Almuntsrbellah M. Almudimeegh, Sulaiman O. AlObaid, Turki H. Albinhar, Muhannad M. Alwadany, Ahmed H. Alajlan, Nora A. Alhedaithi, Razan M. Alawadh, Abdulaziz N. Kadasa, Abdulaziz S. Alobaid, Yazeed H. Alshathry, Rakan H. Alsalhi

**Affiliations:** ^1^ Department of Dermatology College of Medicine, King Saud University Riyadh Saudi Arabia; ^2^ Consultant Dermatology Riyadh Saudi Arabia; ^3^ College of Medicine King Saud University Riyadh Saudi Arabia; ^4^ College of Medicine King Faisal University Hofof Saudi Arabia; ^5^ College of Medicine Princess Nourah Bint Abdulrahman University Riyadh Saudi Arabia; ^6^ College of Medicine Imam Abdulrahman Bin Faisal University Dammam Saudi Arabia; ^7^ College of Medicine King Abdulaziz University Jeddah Saudi Arabia

**Keywords:** gray hair, hair color, risk factors, Saudi Arabia, young adult

## Abstract

**Background:**

Graying is an inherent and unavoidable consequence of the aging process, impacting individuals of all genders. There are limited studies in Saudi Arabia that have examined the prevalence and predictors of premature graying of hair (PGH).

**Objectives:**

This study aims to explore the prevalence and predictors of PGH before the age of 30 among the population of Saudi Arabia.

**Methods:**

This is a cross‐sectional online survey that was conducted between July 2023 and February 2024 in Saudi Arabia. Binary logistic regression analysis was used to identify risk factors of having gray hair before the age of 30.

**Results:**

A total of 1193 participants were involved in this study. A significant portion of respondents reported having gray hair before the age of 30 (55.9%). The younger population (younger than 44 years), smokers, and those who have comorbidities, have anxiety, have depression, have a family history of gray hair before the age of 30 years, have a dry scalp, suffer from vitamin or mineral deficiencies, have hair loss due to immune diseases (such as alopecia), and use minoxidil or rosemary for hair loss were more likely to have gray hair before the age of 30 years (*p* < 0.05).

**Conclusion:**

This study highlighted the high prevalence rate and associated predictors of PGH in Saudi Arabia. Identified predictors include genetic, health, and lifestyle factors. Healthcare professionals and decision makers are advised to promote the awareness of the general public on its risk factors to enhance the prevention of PGH. Public health initiatives include campaigns on smoking cessation, healthy nutrition, and mental health.

## 1 Introduction

Premature graying of hair (PGH) is a complex condition with a range of potential causes. Graying is an inherent and unavoidable consequence of the aging process, impacting individuals of all genders. It arises from a depletion of melanogenically active melanocytes in the hair bulb of gray anagen hair follicles, leading to pigment loss. This pigment loss is a pivotal factor in the pathogenesis of graying. It can be associated with premature aging disorders, atopy, and autoimmune diseases [1]. Where PGH is defined as hair graying occurring before the age of 20 in Caucasians, 25 in Asians, and 30 in Africans [2]. Graying of the hair typically begins at the temples before progressing to the parietal, frontal, and occipital regions, concluding with the occipital region.

In fact, the biological mechanism behind gray hair seems to be linked with the gradual decline of pigment‐producing cells [3]. While hair graying, or canities, is a typical part of the aging process, a portion of individuals undergo premature graying due to familial inheritance or pathological factors [4]. Besides, nutritional deficiencies, particularly in serum calcium, ferritin, and vitamin D3, have been also linked to PGH [5], and other risk factors such as smoking, mineral deficiencies, and certain health conditions have also been identified [6]. However, despite these insights, the exact etiology of PGH remains unclear [1]. Current therapeutic options are limited, while the role of antioxidants and the efficacy of pharmacotherapy are still under investigation [6], and hair dyes are the primary cosmetic solution [1, 7].

A high prevalence of premature graying was found in a sample of 18‐ to 30‐year‐olds in Saudi Arabia, with a significant association with factors such as thyroid issues, vitamin deficiencies, and personal habits [8]. Potentially significant effects of PGH include an individual's diminished self‐esteem, self‐perception, and other psychosocial issues. Indeed, PGH reveals a significant adverse effect on appearance and self‐esteem, which urge the need for effective treatment options [9]. Therefore, this study aims to explore the prevalence and predictors of PGH before the age of 30 among the population of Saudi Arabia. This is essential for comprehending the possible health‐related, environmental, and genetic influences impacting the population. PGH may be associated with several underlying health issues, including oxidative stress, metabolic disorders, and vitamin deficiencies [1], all of which are shaped by prevalent lifestyle and dietary practices in the region. Identification of these factors in advance can help design strategies for awareness, prevention, and improvements in public health.

## 2 Materials and Methods

### 2.1 Study Design and Settings

In Saudi Arabia, a cross‐sectional online survey was conducted between July 2023 and February 2024 to explore the prevalence and predictors of PGH before the age of 30 among the population of Saudi Arabia. This study design is considered a cost‐effective approach to achieve the study's aim within a considerable timeframe.

### 2.2 Sampling Procedure

The sample for this investigation was chosen through a procedure referred to as convenience sampling technique. This particular form of sampling is classified as nonprobability sampling. Patients who satisfied our inclusion criteria and were willing to participate comprised this group. The informed consent form was presented to the participants on the initial page of the questionnaire, where they were also presented with the option to either withdraw from the study or continue their involvement. In order to guarantee patients' understanding of the significance of their involvement, the complete objectives of the research were conveyed. The inclusion criteria were delineated in the invitation letter for the study. We only asked participants who met the inclusion criteria to participate in the study.

### 2.3 Study Population and Recruitment

Constituting the general population, this study's population comprised Saudi Arabia residents aged 18 or older. There are no age or gender restrictions. Encouragement of participation was achieved through the dissemination of the survey link across various social media platforms, including Twitter, Facebook, Instagram, and Snapchat. To encourage participation, we shared the survey link on these networks several times.

### 2.4 Study Tool

The questionnaire tool for this study was developed based on previous literature review. The questionnaire tool had three sections of multiple‐choice questions format and true/false format. The first section asked the participants about their demographic characteristics such as their age, gender, nationality, education level, marital status, employment status, monthly income level, and body mass index (BMI). The second section asked the participants about their sport level (walking, body building, swimming, and running), diet, dietary pattern, whether they take dietary supplement, smoking status, and whether they suffer from any vitamin or mineral deficiency based on the last lab. The third section examined the participants gray hair profile and its associated risk factors. This section asked the participants whether they had gray hair before the age of 30 years, number of gray hairs when appeared, whether they have any comorbidities, whether they have hair loss, type of hair loss, whether they have anxiety or depression, whether they take anxiolytics or antidepressants, type of their scalp, whether they take minoxidil or rosemary for hair loss, whether they have had gray hair after using them, whether they have admitted to hospital before, whether they have viral infection history, whether they use chronic medications, and whether they have family history before the age of 30 years.

### 2.5 Piloting of the Questionnaire Tool

Physicians affiliated with the School of Medicine at King Saud University evaluated and authenticated the questionnaire instrument. The clarity, comprehensibility, and face validity of the queries, as well as whether or not any were challenging to grasp, were evaluated by the participants. Furthermore, participants were queried regarding any inquiries that they perceived as offensive or unpleasant. According to them, the questionnaire was uncomplicated in terms of comprehension and completion. Additionally, prior to the questionnaire's widespread implementation, a pilot study was undertaken to assess the comprehension of the questionnaire among a limited subset of the study population.

### 2.6 Sample Size

The minimum required sample size was 385 individuals using a 95% confidence interval, a 0.5 standard deviation (SD), and a 5% margin of error.

### 2.7 Ethical Approval

This study was approved by the Institutional Review Board at King Saud University, Riyadh, Saudi Arabia.

### 2.8 Statistical Analysis

Using SPSS version 29, this study's data was analyzed. Categorical variables were presented as frequencies and percentages. Chi‐squared test was used to assess whether there is a statistically significance difference in participants' demographic characteristics, lifestyle, and gray hair profile based on their age and PGH status. Binary logistic regression analysis was used to identify risk factors of having gray hair before the age of 30. To determine statistical significance, a two‐sided *p*‐value less than 0.05 was utilized.

## 3 Results

A total of 1193 participants were involved in this study. Males represent 53.0% of the sample, with the majority falling within the age range of 18–29 years. Saudi nationals make up a significant proportion (96.9%) of the respondents. Education‐wise, a large portion hold bachelor's degree (68.3%), followed by secondary‐level education (17.4%). Marital status reveals a diverse distribution, with single individuals comprising 45.2% and married individuals representing 30.4%. In terms of employment, the majority are either students (29.9%) or work in the governmental sector (34.9%). Regarding monthly income, a notable portion earns less than 5000 SAR (36.2%), followed by 6000–10 000 SAR (29.3%) (Table 1).

**TABLE 1:** Participants' demographic characteristics.

Variable	Frequency	Percentage	Frequency	Percentage	Frequency	Percentage	*p*	Frequency	Percentage	Frequency	Percentage	*p*
	Overall (*n* = 1193)		Age group 18–29 (*n* = 687)		Age groups 30 and older (*n* = 506)			Participants with PGH (*n* = 667)		Participants without PGH (*n* = 526)		
*Gender*												
Males	632	53.0%	315	45.9%	317	62.6%	< 0.001	354	53.1%	278	52.9%	0.953
*Nationality*												
Saudi	1156	96.9%	660	96.1%	496	98.0%	0.037	651	97.6%	505	96.0%	0.131
*Education*												
Secondary level	208	17.4%	163	23.7%	45	8.9%	< 0.001	113	16.9%	95	18.1%	0.024*
Diploma	33	2.8%	18	2.6%	15	3.0%		23	3.4%	10	1.9%	
Bachelor degree	815	68.3%	483	70.3%	332	65.6%		446	66.9%	369	70.2%	
Master's degree	103	8.6%	20	2.9%	83	16.4%		70	10.5%	33	6.3%	
PhD	34	2.8%	3	0.4%	31	6.1%		15	2.2%	19	3.6%	
*Marital status*												
Single	539	45.2%	485	70.6%	54	10.7%	< 0.001	286	42.9%	253	48.1%	0.112
Widowed	193	16.2%	74	10.8%	119	23.5%		121	18.1%	72	13.7%	
Divorced	98	8.2%	27	3.9%	71	14.0%		52	7.8%	46	8.7%	
Married	363	30.4%	101	14.7%	262	51.8%		208	31.2%	155	29.5%	
*Employment status*												
Student	356	29.9%	345	50.2%	11	2.2%	< 0.001	174	26.1%	182	34.6%	0.008**
Working in the governmental sector	416	34.9%	168	24.5%	248	49.0%		249	37.4%	167	31.7%	
Working in the private sector	332	27.8%	122	17.8%	210	41.5%		197	29.6%	135	25.7%	
Unemployed	89	7.5%	52	7.6%	37	7.3%		47	7.1%	42	8.0%	
*Monthly income*												
Less than 5000 SAR	432	36.2%	371	54.0%	61	12.1%	< 0.001	224	33.6%	208	39.5%	0.248
6000–10 000 SAR	350	29.3%	165	24.0%	185	36.6%		205	30.7%	145	27.6%	
11 000–20 000 SAR	300	25.1%	114	16.6%	186	36.8%		173	25.9%	127	24.1%	
21 000–30 000 SAR	73	6.1%	30	4.4%	43	8.5%		45	6.7%	28	5.3%	
More than 30 000 SAR	38	3.2%	7	1.0%	31	6.1%		20	3.0%	18	3.4%	

*Note:* In terms of participants' demographic characteristics, all participants' demographic characteristics showed statistically significant difference between participants aged 18–29 years and those aged 30 years and older (*p* < 0.05). Besides, all participants' demographic characteristics showed statistically significant difference based on their PGH status except for gender, nationality, marital status, and monthly income (*p* > 0.05).

* *p* < 0.05.

** *p* < 0.01.

### 3.1 Participants' Lifestyle Profile

Table 2 provides insights into health‐related behaviors and characteristics of the surveyed population. BMI distribution shows that the majority fall within the healthy range, with 60.1% having a BMI between 20 and 25 kg/cm^2^ Regarding physical activity, a significant proportion engage in exercise, with 42.3% participating 2–3 times per week. However, 22.8% report no physical activity. Dietary habits reveal that 57.4% follow a healthy diet, while 42.6% have an unhealthy one. Among those with a dietary pattern, the majority (37.0%) report following a diet program. Additionally, 41.2% take dietary supplements. Smoking is prevalent, with 56.8% identifying as smokers, and among them, a considerable portion (40.6%) smoke on a daily basis. Various types of smoking are reported, with cigarettes being the most common. The majority of smokers suffer from vitamin or mineral deficiencies based on recent lab tests (53.5%). In terms of participants' lifestyle profile, all participants' lifestyle characteristics showed statistically significant difference between participants aged 18–29 years and those aged 30 years and older (*p* < 0.001), except for sport activity level and taking dietary supplement (*p* > 0.05). Similarly, participants' lifestyle profile, all participants' lifestyle characteristics showed statistically significant difference based on participants' PGH status except for BMI and sport activity level (*p* > 0.05).

**TABLE 2:** Participants' lifestyle profile.

Variable	Frequency	Percentage	Frequency	Percentage	Frequency	Percentage	*p*	Frequency	Percentage	Frequency	Percentage	*p*
	Overall (*n* = 1193)		Age group 18–29 (*n* = 687)		Age groups 30 and older (*n* = 506)			Participants with PGH (*n* = 667)		Participants without PGH (*n* = 526)		
*Body mass index category*												
Less than 20 kg/cm^2^	255	21.4%	195	28.4%	60	11.9%	< 0.001	157	23.5%	98	18.6%	0.141
20–25 kg/cm^2^	717	60.1%	367	53.4%	350	69.2%		394	59.0%	323	61.4%	
More than 25 kg/cm^2^	221	18.5%	125	18.2%	96	19.0%		116	17.4%	105	20.0%	
*Sport activity level* (*walking*, *body building*, *swimming*, *running*, *etc*.)												
Once per week	237	19.9%	132	19.2%	105	20.8%	0.138	138	20.7%	99	18.8%	0.804
2–3 times per week	505	42.3%	277	40.3%	227	45.1%		280	41.8%	225	42.8%	
4 times or more per week	179	15.0%	114	16.6%	65	12.85		98	14.6%	81	15.4%	
No activity	272	22.8%	164	23.9%	108	21.3%		151	22.6%	121	23.0%	
*Diet*												
Healthy	685	57.4%	338	49.2%	347	68.6%	< 0.001	411	61.6%	274	52.1%	< 0.001
Unhealthy	508	42.6%	349	50.8%	159	31.4%		256	38.4%	252	47.9%	
*Dietary pattern* (*n = 685*)												
Balanced	227	33.1%	200	52.8%	27	8.8%	< 0.001	120	18.0%	107	20.3%	0.034*
Vegetarian	108	15.8%	76	20.1%	32	10.5%		68	10.2%	40	7.6%	
High protein diet	97	14.1%	76	20.1%	21	6.9%		35	5.2%	62	11.8%	
Following diet program	253	37.0%	27	7.1%	226	73.9%		90	13.5%	163	31.0%	
*Taking dietary supplement*												
Yes	492	41.2%	271	39.4%	221	43.7%	0.080	313	46.9%	179	34.0%	< 0.001
*Smoker*												
Yes	678	56.8%	354	51.5%	324	64.0%	< 0.001	426	63.9%	252	47.9%	< 0.001
*Type of smoking* (*n = 871*)												
Cigarette	375	55.3%	180	37.6%	195	49.7%	< 0.001	239	35.8%	136	25.9%	< 0.001
Electronic cigarette	327	48.2%	203	42.4%	124	31.6%		198	29.7%	129	24.5%	
Hookah	145	21.4%	81	16.9%	64	16.3%		107	16.0%	38	7.2%	
Cigar	24	3.5%	15	3.1%	9	2.3%		13	1.9%	11	2.1%	
*If you are a smoker*, *do you smoke on daily basis?* (*n = 678*)												
Yes	274	40.4%	268	39.1%	126	24.9%	< 0.001	156	23.4%	118	22.4%	< 0.001
*If you smoke on daily basis*, *what is your daily smoke intake?* (*n = 678*)												
Less than half pack per day	195	28.8%	117	17.0%	78	15.4%	< 0.001	129	19.3%	66	12.5%	< 0.001
One pack per day	233	34.4%	95	13.8%	138	27.3%		146	21.9%	87	16.5%	
Less than two packs per day	70	10.3%	23	3.3%	47	9.3%		44	6.6%	26	4.9%	
Two packs or more per day	180	26.5%	80	25.4%	100	27.5%		100	15.0%	80	15.2%	
*Do you suffer from any vitamin or mineral deficiency based on the last lab test you performed?*												
Yes	638	53.5%	400	58.2%	238	47.0%	< 0.001	406	60.9%	232	44.1%	< 0.001

* *p* < 0.05.

### 3.2 Gray Hair Profile

Table 3 presents gray hair profile of the study participants. A significant portion of respondents reported having gray hair before the age of 30 (55.9%), with a majority reporting 1–10 gray hairs (52.5%). Comorbidities were reported by 25.8% of participants, with hair loss prevalent among 53.8% of respondents, primarily attributed to hereditary factors (18.2%). Anxiety and depression were reported by 46.9% and 24.8% of respondents, respectively, with 30.0% of those with anxiety or depression using anxiolytics or antidepressants. Scalp type distribution showed that 51.6% reported having oily scalps. Additionally, 45.2% of respondents used minoxidil for hair loss, with 40.7% reporting gray hair after its use. Rosemary use for hair loss was reported by 54.0% of respondents, with 36.3% reporting gray hair after its use. History of hospital admission was reported by 32.6% of participants. Viral infection history included various types, with COVID‐19 being the most prevalent (80 cases). Chronic medication usage was reported by 25.6% of respondents, including a variety of medications such as immunosuppressants, vitamins, and antihypertensive drugs. A significant proportion (62.2%) reported a family history of gray hair before the age of 30, with fathers being the most commonly mentioned family member (53.4%). In terms of participants' gray hair profile, all participants' gray hair characteristics showed statistically significant difference between participants aged 18–29 years and those aged 30 years and older (*p* < 0.05), except the percentage of participants who had gray hair before the age of 30 years, depressive status, the use of rosemary for hair loss, having hospital admission history, taking chronic medications, and having family history of gray hair before the age of 30 years (*p* > 0.05). Furthermore, participants' gray hair profile showed statistically significant difference between participants based on their PGH status (*p* < 0.001).

**TABLE 3:** Gray hair profile.

Variable	Frequency	Percentage	Frequency	Percentage	Frequency	Percentage	*p*	Frequency	Percentage	Frequency	Percentage	*p*
	Overall (*n* = 1193)		Age group 18–29 (*n* = 687)		Age groups 30 and older (*n* = 506)			Participants with PGH (*n* = 667)		Participants without PGH (*n* = 526)		
*Did you have gray hair before the age of 30 years?*												
Yes	667	55.9%	380	55.3%	287	56.7%	0.336					
*What was the number of gray hairs when?* (*n = 667*)												
1–10	350	52.5%	180	63.8%	170	44.2%	< 0.001	287	43.0%	63	12.0%	< 0.001
11–100	238	35.7%	76	11.1%	162	32.0%		185	27.7%	53	10.1%	
More than 100	79	11.8%	26	3.8%	53	10.5%		48	7.2%	31	5.9%	
*Do you have any comorbidities?*												
Yes	308	25.8%	133	19.4%	175	34.6%	< 0.001	233	34.9%	75	14.3%	< 0.001
*Do you have hair loss?*												
Yes	642	53.8%	400	58.2%	242	47.8%	< 0.001	402	60.3%	240	45.6%	< 0.001
*If yes*, *what is the type of your hair loss?*												
Hereditary	217	18.2%	132	19.2%	85	16.8%	< 0.001	137	20.5%	80	15.2%	< 0.001
Acute (such as after birth hair loss, due to malnutrition)	110	9.2%	66	9.6%	44	8.7%		80	12.0%	30	5.7%	
Due to immune diseases (such as alopecia)	62	5.2%	20	2.9%	42	8.3%		54	8.1%	8	1.5%	
Due to inflammatory or fibrotic diseases of the scalp	33	2.8%	15	2.2%	18	3.6%		25	3.7%	8	1.5%	
Others	205	17.2%	157	22.9%	48	9.5%		94	14.1%	111	21.1%	
*Do you have anxiety?*												
Yes	560	46.9%	357	52.0%	203	40.1%	< 0.001	371	55.6%	189	35.9%	< 0.001
*Do you have depression?*												
Yes	296	24.8%	171	24.9%	125	24.7%	0.498	214	32.1%	82	15.6%	< 0.001
*Do you take anxiolytics or antidepressants?* (*n = 560*)												
Yes	151	30.0%	80	11.6%	71	14.0%	0.040	117	17.5%	34	6.5%	< 0.001
*What is the type of your scalp?*												
Oily	616	51.6%	384	55.9%	232	45.8%	< 0.001	377	56.5%	239	45.4%	< 0.001
Dry	577	48.4%	202	29.4%	157	31.0%		290	43.5%	287	54.6%	
*Did you use minoxidil for hair loss?* (*n = 642*)												
Yes	290	45.2%	142	20.7%	148	29.2%	0.002	222	33.3%	68	12.9%	< 0.001
*If yes*, *did you have gray hair after using it?* (*n = 290*)												
Yes	118	40.7%	52	7.6%	66	13.0%	< 0.001	101	15.1%	17	32.%	< 0.001
*Did you use rosemary for hair loss?* (*n = 642*)												
Yes	347	54.0%	196	28.5%	151	29.8%	0.633	234	35.1%	113	21.5%	< 0.001
*If yes*, *did you have gray hair after using it?* (*n = 347*)												
Yes	126	36.3%	58	8.4%	68	13.4%	0.011	107	16.0%	19	3.6%	< 0.001
*Have you ever been admitted to hospital before?*												
Yes	389	32.6%	219	31.9%	170	33.6%	0.286	252	37.8%	137	26.0%	< 0.001
*Do you have viral infection history?*												
Hepatitis type B	25	2.1%	13	1.9%	12	2.4%	0.009	17	2.5%	8	1.5%	< 0.001
Hepatitis type C	27	2.3%	14	2.1%	13	2.6%		20	3.0%	7	1.3%	
Acquired immune deficiency syndrome	15	1.3%	4	0.6%	11	2.2%		9	1.3%	6	1.1%	
COVID‐19	80	6.7%	16	2.4%	64	12.7%		71	10.6%	9	1.7%	
*Do you take any chronic medications?*												
Yes	306	25.6%	169	24.6%	137	27.1%	0.154	216	32.4%	90	17.1%	< 0.001
*If yes*, *what are these medications?*												
Immunosuppressant (thalidomide, lenalidomide, acitretin, etretinate, prednisone, cyclosporin)	33	2.8%	22	3.2%	11	2.2%	< 0.001	23	3.4%	10	1.9%	< 0.001
Immune‐stimulating medications (erlotinib, latanoprost, tamoxifen, and levodopa, cisplatinum, interferon‐α, and psoralen)	43	3.6%	21	3.1%	22	4.3%		32	4.8%	11	2.1%	
Leprosy medications (clofazimine)	11	0.9%	4	0.6%	7	1.4%		11	1.6%	0	0	
Vitamins (B supplementation with calcium pantothenate or potassium PABA)	78	6.5%	59	8.6%	19	3.8%		52	7.8%	26	4.9%	
Antihypertensive medications	35	2.9%	13	1.9%	22	4.3%		20	3.0%	15	2.9%	
*Do you have gray hair family history before the age of 30 years?*												
Yes	742	62.2%	429	62.4%	313	61.9%	0.441	504	75.6%	238	45.2%	< 0.001
*If yes*, *which family member?* (*n = 742*)												
Father	396	53.4%	199	35.1%	197	44.1%	< 0.001	201	30.1%	195	37.0%	< 0.001
Mother	245	33.0%	121	21.3%	124	27.7%		201	30.1%	44	8.4%	
Brothers or sisters	373	50.3%	247	43.6%	126	28.2%		265	39.7%	108	20.5%	

### 3.3 Predictors of Gray Hair Before the Age of 30 Years

Binary logistic regression analysis identified that the younger population (younger than 44 years), smokers, and those who have comorbidities, have anxiety, have depression, have a family history of gray hair before the age of 30 years, have a dry scalp, suffer from vitamin or mineral deficiencies, have hair loss due to immune diseases (such as alopecia), and use minoxidil or rosemary for hair loss were more likely to have gray hair before the age of 30 years (*p* < 0.05) (Table 4).

**TABLE 4:** Predictors of gray hair before the age of 30 years.

Variable	Odds ratio of having gray hair before the age of 30 years (95% confidence interval)	*p*
*Gender*		
Females	1.00	
Males	0.99 (0.79–1.25)	0.939
*Age category*		
18–29 years	1.00	
30–34 years	1.99 (1.46–2.71)	< 0.001
35–39 years	1.73 (1.26–2.36)	< 0.001
40–44 years	1.54 (1.04–2.27)	0.031*
45–49 years	0.70 (0.30–1.65)	0.415
50–54 years	1.63 (0.65–4.08)	0.296
55–59 years	0.33 (0.11–1.04)	0.059
60 years and older	0.44 (0.13–1.41)	0.165
*Nationality*		
Saudi	1.00	
Non‐Saudi	1.69 (0.87–3.28)	0.119
*Education*		
Bachelor degree	1.00	
Secondary school	0.98 (0.73–1.34)	0.918
Master's degree	0.65 (0.33–1.30)	0.227
PhD	1.76 (0.14–2.72)	0.120
*Marital status*		
Widowed	1.00	
Single	1.49 (0.91–2.43)	0.115
Married	1.00 (0.65–1.53)	1.00
Divorced	1.19 (0.76–1.85)	0.453
*Employment status*		
Student	1.00	
Working in the private sector	0.65 (0.50–0.84)	< 0.001
Working in the governmental sector	0.76 (0.49–1.18)	0.218
Unemployed	—	
*Monthly income*		
Less than 5000 SAR	1.00	
6000–10 000 SAR	0.76 (0.57–1.01)	0.061
11 000–20 000 SAR	0.79 (0.40–1.54)	0.482
21 000–30 000 SAR	0.96 (0.71–1.32)	0.816
More than 30 000 SAR	1.14 (0.68–1.91)	0.627
*Smokers*		
No	1.00	
Yes	1.92 (1.52–2.43)	< 0.001
*Do you have any comorbidities?*		
No	1.00	
Yes	3.23 (2.41–4.32)	< 0.001
*Do you have anxiety?*		
No	1.00	
Yes	2.24 (1.77–2.83)	< 0.001
*Do you have depression?*		
No	1.00	
Yes	2.56 (1.92–3.41)	< 0.001
*Type of your scalp?*		
Dry	1.00	
Oily	0.39 (0.28–0.54)	< 0.001
*Do you have gray hair family history before the age of 30 years?*		
No	1.00	
Yes	3.74 (2.93–4.78)	< 0.001
*Body mass index category*		
Less than 20 kg/cm^2^	1.00	
20–25 kg/cm^2^	0.76 (0.57–1.02)	0.065
More than 25 kg/cm^2^	0.70 (0.48–1.00)	0.051
*Sport activity level*		
No activity	1.00	
Once per week	1.04 (0.71–1.52)	0.831
2–3 times per week	1.16 (0.79–1.72)	0.448
4 times or more per week	1.04 (0.74–1.46)	0.842
*Diet*		
Unhealthy	1.00	
Healthy	0.68 (0.54–0.85)	< 0.001
*Dietary pattern*		
Balanced	1.00	
Vegetarian	1.32 (0.92–1.89)	0.129
High protein diet	1.05 (0.73–1.51)	0.806
Following diet program	0.94 (0.55–1.60)	0.805
*Taking dietary supplement*		
No	1.00	
Yes	1.72 (1.36–2.18)	< 0.001
*Type of smoking*		
Cigarette	1.00	
Electronic cigarette	0.79 (0.55–1.15)	0.223
Hookah	1.12 (0.73–1.73)	0.607
Cigar	3.96 (0.48–32.72)	0.201
*If you are a smoker*, *do you smoke on daily basis?*		
No	1.00	
Yes	0.95 (0.70–1.29)	0.738
*If you smoke on daily basis*, *what is your daily smoke intake?*		
Less than half pack per day	1.00	
One pack per day	0.86 (0.58–1.28)	0.453
Less than two packs per day	0.87 (0.49–1.53)	0.619
Two packs or more per day	0.63 (0.25–1.58)	0.322
*Do you suffer from any vitamin or mineral deficiency based on the last lab test you performed?*		
No	1.00	
Yes	1.99 (1.58–2.51)	< 0.001
*Do you have hair loss?*		
No	1.00	
Yes	1.81 (1.43–2.28)	< 0.001
*If yes*, *what is the type of your hair loss?*		
Hereditary	1.00	
Acute (such as after birth hair loss, due to malnutrition)	1.56 (0.94–2.57)	0.084
Due to immune diseases (such as alopecia)	3.94 (1.79–8.70)	< 0.001
Due to inflammatory or fibrotic diseases of the scalp	1.83 (0.79–4.24)	0.162
Others	0.50 (0.34–0.73)	< 0.001
*Did you use minoxidil for hair loss?*		
No	1.00	
Yes	3.38 (2.50–4.57)	< 0.001
*Did you use rosemary for hair loss?*		
No	1.00	
Yes	1.99 (1.53–2.59)	< 0.001
*Do you have viral infection history?*		
Hepatitis type B	1.00	
Hepatitis type C	0.85 (0.08–9.30)	0.895
Acquired immune deficiency syndrome	0.11 (0.00–3.53)	0.213
COVID‐19	0.18 (0.02–1.41)	0.101

* *p* < 0.05.

## 4 Discussion

Hair graying results from gradual decline of pigment‐producing cells, and it is called PGH when it occurs before the age of 30 [4]. Indeed, it is a multifaceted condition with various possible underlying factors [1]. Therefore, this study aimed to explore the prevalence of PGH and its associated predictors before the age of 30 among the population of Saudi Arabia.

The study results found that a significant portion of respondents reported having gray hair before the age of 30 (55.9%), with a majority reporting 1–10 gray hairs (52.5%). This represents a substantially high prevalence of PGH before the age of 30 among the sample. In fact, these findings are higher than the findings from other studies, where in Islamabad 31.2% was the prevalence of PGH among medical student, [10] and 27.3% among the young adults of Indian population, [11] and PGH is considered as uncommon in Nigeria [12]. This difference of prevalence is most likely due to the variations of risk factors that cause the incidence of PGH, including but not limited to hereditary factors, smoking, and diet [6]. Two forms of melanin are present in human hair follicles: eumelanin and pheomelanin [1]. The quantity and ratio of black‐brown eumelanin and reddish‐brown pheomelanin are the primary factors contributing to the diversity of hair color. The phenotype of hair is believed to be influenced by the pH and cysteine level of melanosomes [1]. The auburn or red color of hair is the result of a mutation in the melanocortin‐1 receptor (MC1R) gene. This mutation is typically observed in individuals from Northern Europe who receive less sunlight [13, 14]. In 2012, a recessive mutation in tyrosinase‐related protein 1 (TYRP1) was identified in individuals with blonde hair [15]. Melanin production, therefore, the pigmentation of hair, is under the influence of many factors: changes related to the hair cycle, sex and racial differences, hormonal status, genetic background, and last but not least age‐related changes [16]. Other exogenous factors may hasten the graying process, namely ultraviolet (UV) radiation and nutritional status [17]. The average age of onset of hair graying seems to be the mid‐ to late 40s, although this is modified by race: for Caucasians, it is around the mid‐30s; for Asians, late 30s; and for Africans, mid‐40s [18].

Individuals in Saudi Arabia are exposed to higher UV radiations compared to the populations in temperate climatic zones [19]. The exposure is higher due to the high solar radiation in the region. Saudi Arabian climate is a desert climate with clear skies and, therefore, bright sun throughout most part of the year, which results in great exposure to UV rays [19]. The intensity of sunshine and geographical location make the biggest difference with regard to UV exposure. Generally, the intensity of UV radiation is higher in equatorial and desert regions, such as Saudi Arabia, because it receives direct sunlight throughout the year. High exposure to UV may result in several skin conditions, such as premature skin aging [1].

The study result found that 25.8% of participants reported having comorbidities, with 53.8% of respondents experiencing hair loss, primarily attributed to hereditary factors (18.2%). Furthermore, 25.6% of respondents reported chronic medication usage, encompassing a range of medications like immunosuppressants, vitamins, and antihypertensive drugs. A notable proportion (62.2%) reported a family history of gray hair before the age of 30, with fathers being the most frequently mentioned family member (53.4%). In fact, PGH was found to be associated with many comorbidities including premature cardiovascular disease [20] and mineral deficiencies [6]. On the other hand, it was wildly reported that PGH has hereditary and genetic influence on prevalence of PGH in individuals aged less than 30 [4, 6, 21]. Family history of PGH is a significant risk factor associated with premature graying in young adults [21]. Furthermore, it was found that waist circumference, systolic and diastolic blood pressures, and fasting blood sugar were higher in people with PGH [22]. In a recent study conducted on young adults in Turkey, it was found that PGH is closely associated with oxidative stress‐causing factors, including emotional stress and chronic diseases, in genetically predisposed men and women [23]. The Global Burden of Disease country‐specific report showed that the high prevalence of chronic diseases in Saudi Arabia was the primary cause of death in the country. Obesity, diabetes mellitus, and hypertension were the most common comorbidities among the Saudi population [24].

Moreover, 46.9% of respondents reported experiencing anxiety, and 24.8% reported depression. Among those with anxiety or depression, 30.0% reported using anxiolytics or antidepressants. Indeed, PGH has been associated with a range of factors, including family history of the condition, iron deficiency, and a history of depression, [25] where the psychological impact of this condition is significant, with individuals experiencing low self‐esteem and potential socio‐cultural challenges [9]. On the other hand, using anxiolytics or antidepressants is associated with hair loss that potentially contribute to PGH [26]. However, the exact mechanism of this association is not yet fully understood. A previous study conducted on young adults in Turkey demonstrated that PGH is closely associated with factors that induce oxidative stress, including emotional stress [23]. A previous study in Saudi Arabia found high prevalence rate of depression (12.7%) and anxiety (12.4%). This was accompanied by low diagnosis rate [27].

In our study, scalp type distribution showed that 51.6% reported having oily scalps. Additionally, 45.2% of respondents used minoxidil for hair loss, with 40.7% reporting gray hair after its use. Rosemary use for hair loss was reported by 54.0% of respondents, with 36.3% reporting gray hair after its use. In fact, it is suggested that a reduction in melanogenically active melanocytes in the hair bulb, defective melanosomal transfers, and melanin incontinence contribute to the PGH process, [9] where this is the most suggested theory for the etiology of PGH. Although minoxidil or rosemary is used to promote hair growth especially for the treatment of alopecia [28, 29], current research does not support their effectiveness as a leading cause, preventive, or treatment for PGH.

In addition, viral infection history included various types, with COVID‐19 being the most prevalent risk factor for related to PGH. It was found that viral infections especially COVID‐19 may lead to hair loss and its related conditions including alopecia due to certain factors, where the more severe the infection, the more profound and prolonged the course of alopecia [30]. Also, it is known that viral infection may lead to nutrient depletion [31], which may play as causing factor for PGH [5].

The study results identified that the younger population (younger than 44 years), smokers, and those who have comorbidities, have anxiety, have depression, have gray hair family history before the age of 30 years, have a dry scalp, suffer from vitamin or mineral deficiencies, have hair loss due to immune diseases (such as alopecia), and use minoxidil or rosemary for hair loss were more likely to have gray hair before the age of 30 years. PGH is determined by an interplay of genetic predispositions, lifestyle, and various health‐related factors. Those who smoke are at an elevated risk, attributable to the elevated oxidative stress and the harm to melanocytes that arises from tobacco consumption [32]. Furthermore, individuals suffering from comorbid conditions may experience impaired melanin synthesis, resulting in PGH [1, 6]. Anxiety and depression have the potential to increase cortisol levels, which adversely affects hair pigmentation [25]. A familial background of PGH significantly indicates a genetic vulnerability, given that genetic research has pinpointed particular loci linked to hair color [33]. In addition, a dry scalp may lead to poor scalp health and reduced nutrition to the hair follicles, which can also contribute to gray hair [1].

The nutritional deficiencies of essential vitamins and minerals, such as iron and zinc, have been linked to hair pigmentation in view of their critical role in melanocytes' activities and overall hair health [34]. Immune diseases, such as alopecia, result in hair loss and disturb the normal pattern of hair regrowth, and thus, the melanocytes may malfunction and cause early graying [34]. The application of hair loss therapies, including minoxidil and rosemary, is intended to facilitate the regrowth of hair; however, these interventions do not address the issue of hair graying. Specifically, minoxidil predominantly activates hair follicles without exerting any direct influence on hair pigmentation, resulting in the possibility that newly regrown hair may retain a gray [35]. Additionally, although rosemary is utilized for its antioxidant benefits, there is little evidence supporting its efficacy in directly influencing hair color.

At the same time, our study findings found that younger population (younger than 44 years) were more likely to report that they had PGH before the age of 30 compared to the older age groups. Besides, those working in the private sector and those with oily scalp were less likely to report PGH. Younger population are exposed to more environmental and lifestyle risk factors; for instance, elevated levels of stress and dietary deficiencies may be involved in the susceptibility to PGH. As concerns private employees, these subjects show a lower risk to develop PGH. This may concern the particular characteristics of their work or the level of stress they experience. Private sector employees may face a very competitive and frantic environment, which may push them to healthier lifestyles or cosmetic corrections to cope with PGH, hence, lowering the likelihood of developing PGH. In the end, it is unlikely that people with oily scalps will complain of PGH, as good hydration of the scalp generally favors full‐spectrum health of hair, including growth rate and maintenance of melanin, a sign of no graying of hair. It could mean that the sebaceous gland system is healthy; such a system protects the hair follicle from damage to prevent gray hair. Conversely, a dry scalp has persistently been associated with hair damage and oxidative stress, which actually accelerates the process of graying [1]. Therefore, healthcare providers in Saudi Arabia should educate high‐risk individuals, particularly younger populations, smokers, and those with comorbidities, about the multifactorial nature of PGH. Lifestyle changes such as quitting smoking, maintaining a balanced diet, and managing stress can help mitigate PGH risk factors. Routine screening for comorbidities and psychological well‐being is essential for early intervention. Adherence to preventive measures against viral infections, including COVID‐19, is crucial.

This study has limitations. Due to the cross‐sectional study design, we were unable to examine causality among the study variables. The use of an online survey might have missed some of our targeted populations who are not using social media platforms. As we used convenience sampling technique using an anonymized online survey link, we were not able to estimate the number of participants who had received the study link. Therefore, we are unable to estimate the response rate for our study, which has affected the generalizability of our findings. Moreover, online survey study design is subject to reporting bias, recall bias, and social desirability bias. These types of bias might arise for several reasons, including when participants inaccurately recall past events or experiences, when they misunderstand questions or prefer not to disclose sensitive information, and when respondents respond to questions in a manner, they believe is socially acceptable rather than truthfully. Participants' medical diagnosis, lab tests findings, depressive and anxiety status were examined by asking them directly without clinical diagnosis, which might have led to misdiagnosis. Therefore, our study findings should be interpreted carefully.

In conclusion, multiple factors, including family history, comorbidities, anxiety, depression, and scalp type, considerably contribute to the alarming prevalence of PGH among the respondents of this study. Younger populations (44 years and younger), smokers, and individuals with comorbidities including depression, anxiety, and premature graying in the family, as well as those who smoke, are at greater risk for the consequences of early detection and intervention. Additional investigation is warranted to clarify the fundamental mechanisms that connect these variables with premature graying, to examine therapeutic approaches that have fewer adverse effects, and to design customized interventions specifically for populations that are at increased risk. It is imperative that healthcare professionals deliver comprehensive management approaches that address both esthetic concerns and the accompanying psychological distress while placing screening for these risk factors as a clinical priority.

## Author Contributions

**A.M.A.** and **S.O.A.:** supervised the study. **A.M.A., S.O.A., T.H.A., M.M.A., A.H.A., N.A.A., R.M.A., A.N.K., A.S.A., Y.H.A.**, and **R.H.A.:** designed the study. **T.H.A., M.M.A., A.H.A., N.A.A., R.M.A., A.N.K., A.S.A., Y.H.A.**, and **R.H.A.:** collected and analyzed the data. **A.M.A., S.O.A., T.H.A., M.M.A., A.H.A., N.A.A., R.M.A., A.N.K., A.S.A., Y.H.A.**, and **R.H.A.:** participated in writing the manuscript. **A.M.A., S.O.A., T.H.A., M.M.A., A.H.A.**, and **N.A.A.:** read and approved the final manuscript.

## Ethics Statement

Approval (No. E‐23‐1259) was obtained from the Institutional Review Board of the College of Medicine, King Saud University, Riyadh, SA. Each participant approved through an online questionnaire after being informed about the study's purpose and their right to withdraw at any time without obligation to the study team. Furthermore, participant anonymity was ensured by refraining from collecting identifying data. No incentives or rewards were provided to the participants.

## Conflicts of Interest

The authors declare no conflicts of interest.

## Supporting information


**Data S1:** Questionnaire tool.

## Data Availability

The data that support the findings of this study are available from the corresponding author upon reasonable request.
