# Supplementary material for: Prevalence and Predictors of Premature Graying of Hair Before the Age of 30: A Cross‐Sectional Study in Saudi Arabia
Source: J Cosmet Dermatol. 2024 Oct 9;24(1):e16627. doi: 10.1111/jocd.16627 (PMC11743289; doi:10.1111/jocd.16627)
Supplement: Supplementary file 1 — Data S1: Questionnaire tool. [file JOCD-24-e16627-s001.docx]

**Sociodemographic data:**

1. Gender

- Male
- Female

1. Nationality

- Saudi
- Non-Saudi

1. Age

- 18-24
- 25-29
- 30-34
- 35-39
- 40-44
- 45-49
- 50-54
- 55-59
- More than 60

1. Level of education

- Secondary
- Diploma
- Bachelors degree
- Masters degree
- PhD

1. Marital status

- Single
- Widowed
- Divorced
- Married

1. Occupation

- Student
- Working in the government sector
- Working in the private sector
- Unemployed

1. Socioeconomic status

- Less than 5 thousand riyals
- From 6 to 10 thousand riyals
- From 11 to 20 thousand riyals
- From 21 to 30 thousand riyals
- More than 30 thousand riyals

**Participants lifestyle profile**

1. BMI (you can calculate it using this link <https://www.moh.gov.bh/HealthInfo/BMI>)

- Less than 20 kg/cm^2^
- From 20-25 kg/cm^2^
- More than 25 kg/cm^2^

1. Level of sport activity (ex: walking, body building, swimming, running…etc)

- Once per week
- 2-3 times per week
- 4 times or more a week
- No activity

1. Diet

- Healthy
- Non-healthy

1. If your diet is healthy, what’s your dietary pattern ?

- Balanced
- Vegetarian
- High protein
- Following diet program

1. Do you take any dietary supplements ?

- Yes
- No

1. Are you a smoker ?

- Yes
- No

1. What type of cigarettes do you use ? (you can answer multiple answers)

- Cigarettes
- Electronic cigarettes
- Hookah
- Cigars

1. If you are a smoker, do you smoke everyday ?

- Yes
- No

1. If you are a smoker, how many cigarettes do you consume on a daily basis ?

- Less than half a pack per day
- One pack per day
- Less than two packs per day
- Two packs or more per day

1. Do you consume alcohol and if so how many times per week ?

- Once or twice per week
- More than twice a week
- Once a month
- I don’t consume alcohol

1. Do you suffer from any vitamin or mineral deficiency based on the last lab test you performed?

- Yes
- No

1. If yes, which vitamins/minerals do you have a deficiency in ?

- Zinc deficiency
- Calcium deficiency
- Biotin deficiency
- Vitamin D deficiency
- B1 (thiamine) deficiency
- B3 deficiency
- B5 deficiency
- B6 deficiency
- B12 deficiency
- Vitamin C deficiency
- Ferritin deficiency
- Folic acid deficiency
- Vitamin E deficiency
- Copper deficiency

**Personal history**

1. Do you have grey hair before the age of 30 years ?

- Yes
- No

1. If yes, which age did grey hairs appear:
2. If no, which age did grey hairs appear:
3. When grey hair appeared, how many numbers or grey hairs where there?

- 1-10 hairs
- 11-100 hairs
- More than 100 hairs

1. Do you have any comorbidities?

- Yes
- No

1. If yes, which of these chronic illnesses do you have ?

- Hypertension
- Diabetes mellitus
- Hyper/hypothyroidism
- Immunodeficiency diseases (vitiligo, alopecia…etc)
- Dyslipidemia
- Inherited disorders (neurofibromatosis, tuberous sclerosis…etc)

1. Do you suffer from hair loss ?

- Yes
- No

1. If yes, what type of hair loss do you suffer from ?

- Hereditary
- Acute (postpartum, poor nutrition…etc)
- Due to immune diseases (alopecia, SLE…etc)
- Due to inflammatory or fibrotic diseases of the scalp (Lichen planopilaris..etc)
- Others

1. Do you have anxiety ?

- Yes
- No

1. Do you have depression ?

- Yes
- No

1. Do you take any medications for anxiety/depression ?

- Yes
- No

1. What type of scalp do you have ?

- Oily
- Dry

1. Have you used (Minoxidil) on your scalp for hairloss ?

- Yes
- No

1. If yes, did you have grey hair after using it ?

- Yes
- No

1. Have you used (Rosemary oil) on your scalp ?

- Yes
- No

1. If yes, did you have grey hair after using it ?

- Yes
- No

1. Have you ever been admitted to the hospital before ?

- Yes
- No

1. Do have vital infection history of any of the following ?

- Hepatitis B virus
- Hepatitis C virus
- Acquired immunodeficiency
- Covid-19 (corona virus)

1. Do you take any chronic medications ?

- Yes
- No

1. If yes, what are these medications (you can choose multiple answers) ?

- Immunosuppressants ( thalidomide , lenalidomide . acitretin ،etretinate، prednisone, cyclosporin)
- Immune stimulating medication (erlotinib ، latanoprost, tamoxifen, and levodopa, cisplatinum, interferon-α, and psoralen)
- Leprosy medications (Clofazimine)
- Vitamins (B supplementation with calcium pantothenate or potassium PABA)
- Hypertension medication (Captopril)

**Family history**

1. Do you have grey hair family history before the age of thirty ?

- Yes
- No

1. If yes, which family members (you can choose multiple answers) ?

- Father
- Mother
- Siblings (brothers/sisters)
